# The transmissibility of noroviruses: Statistical modeling of outbreak events with known route of transmission in Japan

**DOI:** 10.1371/journal.pone.0173996

**Published:** 2017-03-15

**Authors:** Ryota Matsuyama, Fuminari Miura, Hiroshi Nishiura

**Affiliations:** 1 Graduate School of Medicine, Hokkaido University, Kita 15 Jo Nishi 7 Chome, Kita-ku, Sapporo, Japan; 2 CREST, Japan Science and Technology Agency, 4-1-8, Honcho, Kawaguchi-shi, Saitama, Japan; 3 Department of Urban Engineering, Graduate School of Engineering, the University of Tokyo, 7-3-1 Hongo, Bunkyo-ku, Tokyo, Japan; University of Michigan, USA, UNITED STATES

## Abstract

In Japan, the fraction of norovirus outbreaks attributable to human-to-human transmission has increased with time, and the timing of the increased fraction has coincided with the increase in the observed fraction of genogroup II genotype 4 (GII.4). The present study aimed to estimate the time-dependent changes in the transmissibility of noroviruses. The effective reproduction number (*R*_*y*_), for year *y*, was estimated by analyzing the time series surveillance data for outbreak events from 2000 to 2016. *R*_y_ was estimated by using the fraction of outbreak events that were attributable to human-to-human transmission and by employing three different statistical models that are considered to mechanistically capture the possible data-generating process in different ways. The *R*_*y*_ estimates ranged from 0.14 to 4.15 in value, revealing an overall increasing trend (p<0.05 for all three models). The proportion of outbreaks caused by GII and GII.4 viruses among the total events also increased with time, and positive correlations were identified between transmissibility and these proportions. Parametric modeling of *R*_y_ indicated that the time-dependent patterns of *R*_y_ were better described by a step function plus linear trend rather than the step function alone that reflects the widespread use of reverse transcriptase PCR (RT-PCR) in and after 2007 for laboratory diagnosis. Accordingly, we conclude that norovirus transmissibility has increased over the past 16 years in Japan. The change is at least partially explained by the time-dependent domination of the contagious GII genogroup (e.g., GII.4), indicating that noroviruses better fitted to humans have selectively caused the human-to-human transmissions, thereby altering the epidemiology of this pathogen.

## Introduction

Norovirus, a member of the *Caliciviridae* family, is a positive-sense single-stranded RNA virus. The virus is known to be a major causal agent of acute gastroenteritis in humans, especially during the winter season. Outbreaks of norovirus infection (NVI) have huge societal and health care costs globally because the virus has a strong infectious nature and perceived acute symptoms shortly after illness onset [1,2]. The major mode of NVI transmission includes foodborne as well as human-to-human transmission via fecal-oral and vomit-oral routes. The median infective dose is as small as 18 to 10^3^ virus particles [3], and viruses remain stable in the environment [4]. Since norovirus was first recognized, possible interventions to prevent secondary transmissions with it have been sought, but controlling it is difficult because of its speed of infection, the mild, nonspecific symptoms it causes, and its multiple transmission routes.

As part of the evolutionary process, viruses change their pathogenesis, virulence and transmissibility characteristics. As seen in the evolution of influenza virus [5], the multistrain dynamics of norovirus is considered to involve antigenic drift (i.e., changes in epitopes within the capsid P2 domain) and shift (e.g., overlapping recombination in open reading frame, ORF 1, and ORF 2), which allows the virus to escape the pressure of human immunity [6–9]. For instance, genogroup II (GII) and genotype 4 (GII.4) strains have caused at least six global epidemics [10–15] during the past 20 years, and have experienced antigenic evolution [6,7]. The newly emerged genotype GII.17 is no exception in this context: it has been the predominant strain in a number of recent disease outbreaks [16–18].

Antigenic evolution most likely involves evolutionary changes in virus transmissibility. To assess such transmissibility, the reproduction number (i.e., the average number of secondary infections generated by one primary case) hereafter called *R*_*y*_, is an indispensable and representative epidemiological measurement. *R*_y_ does not dynamically change in a finer time scale than year, and in every single year the interpretations of *R*_y_ are analogous to those of *R*_0_, except the fact that *R*_y_ is assumed to be able to vary by years. When it comes to the transmissibility of NVIs, *R*_*y*_ has been estimated in a few published studies, where the estimated basic reproduction number (*R*_0_) broadly ranged from 2–18 in closed or semi-closed environments [19–23]. However, the transmissibility has yet to be estimated for an entire human population and, notably, we have yet to explore any possible signature of time-dependent changes in the *R*_*y*_ of NVI to decipher the transmissibility.

Therefore, in the present study, we have explored whether a time-dependent signature exists for norovirus transmissibility by investigating the epidemiological dataset for Japan [24]. Our purpose was to estimate the *R*_*y*_ for NVI as a function of time using the national surveillance data and employing a few parsimonious mathematical models. We also explored the possible impact of the predominance of genogroup GII and genotype GII.4 on the time-dependent change in NVI transmissibility. These study objectives were accomplished using the following methods and data.

## Materials and methods

### Data source

In Japan, NVI outbreak events with confirmed virus detection are notified via the prefectural institutes of public health to the National Institute of Infectious Diseases as part of the National Epidemiological Surveillance of Infectious Diseases (NESID; http://www.nih.go.jp/niid/ja/iasr-noro.html) requirements [24]. The present study used the time series of the notified outbreak events (i.e., the count data of outbreak events) from September 2000 to August 2016 across Japan. When an outbreak is notified via the prefectural institute of public health, the notification document includes whether the outbreak was foodborne or mainly caused by human-to-human transmission. The notification system trusts prefectural institutes to judge whether the outbreak involves human-to-human transmission or is foodborne based on the epidemiological investigations, and if the cause remains unclear even after an epidemiological investigation, the outbreak events are reported as cause “unknown”. In many instances, foodborne outbreaks (attributed to “environmental” exposure) are notified to this surveillance due to the need to virologically verify that a foodborne outbreak was caused by noroviruses, because Food Hygiene Law enforces the suspension of business at any restaurant or other eating facilities with norovirus outbreaks. Because the type of virus responsible for the outbreak is typed using the detected virus, the notification data also contains genogroup and genotype information. Also, because the notification relies on virological confirmation, the absolute number of outbreak events will be under stated, but here we assume that the proportion of observed foodborne outbreaks among all notified outbreaks reflects the unbiased proportion. Potential ascertainment biases are examined in sensitivity analysis.

### Mathematical model

**Fig 1:**
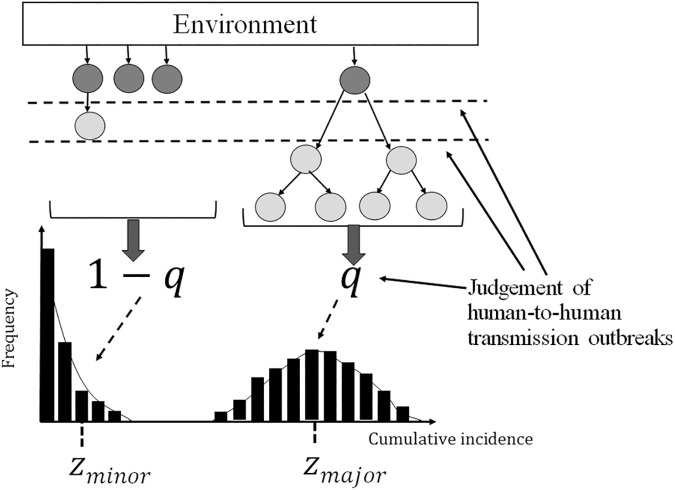
The data-generating process used for investigating norovirus outbreak events. Foodborne cases (dark gray) generate secondary cases via human-to-human transmissions (light gray). Given the final size distribution, let *q* be the proportion of major epidemics among the total environmental exposures. The expected final sizes of the major and minor outbreaks are expressed as *z*_*major*_ and *z*_*minor*_, respectively. Judgment of a human-to-human transmission outbreak is considered to have taken place based on the presence of chains of secondary transmissions (i.e. major epidemic), the presence of at least one secondary case, or simply the random sampling result from the final size.

Fig 1 illustrates our proposed model building. Once a person is environmentally exposed to norovirus (i.e., via shellfish or other contaminated foods), index case(s) are classified as foodborne NVIs. Some of the foodborne NVIs decline to extinction, but the remainder involve chains of human-to-human secondary transmissions with *R*_*y*_ in year *y* (hereafter, we employ a piecewise constant reproduction number by 1 year). If the value of *R*_*y*_ is very large, a certain number of secondary, tertiary and further cases may occur. In such a situation, the majority of cases are caused by human-to-human transmission. In contrast, foodborne cases will be in the majority if a large number of individuals acquire NVIs directly from contaminated foods and if very few human-to-human transmissions follow. The judgment at the prefectural institute of public health regarding the most plausible route of transmission (i.e., whether the outbreak event was foodborne or human-to-human) must take place by investigating the following: (i) the presence of a common source of infection (e.g., common food items) among the diagnosed cases and, (ii) the contact history among the cases during the outbreak investigation (Fig 1). It is not surprising that the *R*_*y*_ mirrors the fraction of human-to-human transmission events among the total.

Here, we have built a parsimonious statistical model to describe the data-generating process and estimate the *R*_y_ from the dataset. Let *q* be the proportion of the human-to-human transmission events among the total of outbreak events. Because *q* is interpreted in several different ways, we built three different models to capture the data-generating process of each interpretation scenario. In each model, the following assumptions were made. (1) We disregard a partly immunized heterogeneous population due to exposures in earlier years and any herd immunity effects are reflected in the estimate of *R*_y_ (in a homogeneous manner). (2) Heterogeneous susceptibility and contact are ignored. (3) An individual experiences one exposure event per year at most (i.e. our model does not account for multiple infection events with noroviruses in a year). (4) Ascertainment bias of cases does not affect the fraction of foodborne NVI outbreak events among the total notified events (i.e., the ascertainment process is independent of the reporting process of the major route of transmission). (5) Index cases were environmentally transmitted (e.g. can be attributed to a responsible food item).

#### Interpretation scenario 1: Reported outbreaks are the result of random sampling of the outbreak events

Let *q* be the proportion of major outbreaks among the total environmental exposure events. In this scenario, we assume that the observed fraction of human-to-human transmission outbreak events mirrors the fraction of major epidemics in the final size distribution. Assuming that the generation time of human-to-human transmission of norovirus is exponentially distributed, we have *q* = 1-1/*R*_y_ given one index case [25], because the probability of extinction is given by 1/ *R*_*y*_. Suppose that *n*_y_ outbreak events were foodborne among a total of (*n*_y_+*m*_y_) outbreaks in year *y*, assuming that observation of the fraction of foodborne outbreaks is approximated by a binomial sampling process, the likelihood function to estimate *R*_y_ is

$$L(R_{y};n_{y},m_{y})=\left(\begin{array}{c} n_{y}+m_{y}\\ n_{y} \end{array}\right){\left(\frac{1}{R_{y}}\right)}^{n_{y}}{\left(1-\frac{1}{R_{y}}\right)}^{m_{y}}$$ (1)

Note that this scenario requires an assumption of *R*_y_>1.

#### Interpretation scenario 2: Reported outbreaks are the result of random sampling of the patients

In this scenario, we assume that the observed fraction of human-to-human transmission outbreak events mirrors the fraction of cases that were involved in major epidemics. Hereafter, a “major epidemic” refers to a large epidemic that does not stochastically decline to extinction without depletion of susceptibles or successful control, while a “minor outbreak” represents a small scale occurrence of cases that is stochastically terminated. The average outbreak size differs between the foodborne and the human-to-human transmission outbreak events. Assuming that the observed fraction of human-to-human transmission outbreak events among the total events reflects the fraction of cases attributable to human-to-human transmissions, the final sizes of the minor and major outbreaks appear to be useful. Assuming that the generation time is exponentially distributed, and thus, the offspring distribution follows a geometric distribution [26], the final size of a minor outbreak (*z*_*minor*_) is

$$z_{minor}=\left(\frac{R_{y}}{R_{y}-1}\right)\frac{1}{N},$$ (2)

where *N* is the population size. The derivation of (2) is discussed in S1 Methods. The final size of a major outbreak (*z*_*major*_) satisfies

$$1-z_{major}=\exp(-z_{major}R_{y})$$ (3)

Employing a second-order Taylor series approximation, we get a closed form solution of *z*_*major*_:

$$z_{major}=\frac{2(R_{y}-1)}{R_{y}{}^{2}}.$$ (4)

Using (2) and (4), the likelihood function to estimate *R*_y_ is

$$L(R_{y};n_{y},m_{y})=\left(\begin{array}{c} n_{y}+m_{y}\\ n_{y} \end{array}\right){\left(\frac{\frac{1}{R_{y}}\left(\frac{R_{y}}{R_{y}-1}\right)\frac{1}{N}}{\frac{1}{R_{y}}\left(\frac{R_{y}}{R_{y}-1}\right)\frac{1}{N}+\left(1-\frac{1}{R_{y}}\right)\left(\frac{2(R_{y}-1)}{R_{y}{}^{2}}\right)}\right)}^{n_{y}}{\left(\frac{\left(1-\frac{1}{R_{y}}\right)\left(\frac{2(R_{y}-1)}{R_{y}{}^{2}}\right)}{\frac{1}{R_{y}}\left(\frac{R_{y}}{R_{y}-1}\right)\frac{1}{N}+\left(1-\frac{1}{R_{y}}\right)\left(\frac{2(R_{y}-1)}{R_{y}{}^{2}}\right)}\right)}^{m_{y}}$$ (5)

#### Interpretation scenario 3: Reported fractions of human-to-human outbreak events reflect the presence of at least one secondary transmission event given an environmental exposure

In this scenario, we consider the possibility that any secondary transmission event from an index case may result in the outbreak notification recorded as arising mainly from human-to-human transmission. In such an instance, the frequency of foodborne infections is the probability of extinction within one generation (i.e., the absence of secondary transmission). Assuming again that the offspring distribution is geometrically distributed, the conditional probability of extinction within one generation is 1/(1 + *R*_*y*_) (see S1 Methods). Accordingly, the likelihood function to estimate *R*_y_ in this scenario is

$$L(R_{y};n_{y},m_{y})=\left(\begin{array}{c} n_{y}+m_{y}\\ n_{y} \end{array}\right){\left(\frac{1}{1+R_{y}}\right)}^{n_{y}}{\left(\frac{R_{y}}{1+R_{y}}\right)}^{m_{y}}$$ (6)

Note that this scenario does not impose any assumption of criticality, that is, *R*_y_ can be either smaller or greater than the value of 1.

Maximum likelihood estimates of *R*_*y*_ were obtained by minimizing the negative logarithm of the above mentioned likelihood functions. The 95% confidence intervals (CI) were derived from the profile likelihood. To compare goodness-of-fit among all scenarios, we calculated Akaike’s information criterion (AIC). To assess the presence of the time-dependent trend in the *R*_y_ estimates, a test of trend for a continuous variable (i.e., Jonckheere-Terpstra test) was employed. We also explored possible correlations between *R*_*y*_ and the proportions of GII and GII.4 among the total of reported virus detections. Pearson’s moment correlation was employed for determining the correlation.

Moreover, to interpret estimates of *R*_y_ in relation to an associated time event, we have also decomposed the time-dependent patterns of *R*_y_ into one or more interpretable components. That is, the use of real-time polymerase chain reaction (RT-PCR) was initiated in 2001 in Japan and there has been a revision to the 2003 guidelines. For this reason, the use of RT-PCR has become widespread in and after the year 2007, potentially improving laboratory detection around that year. If the proportion of a certain genotype (e.g. GII.4) is caused by that change, the estimated *R*_y_ that rests on the observed proportion should be approximated by

$$R_{y}=\left\{ \begin{array}{l} k,\;\text{for}\;y\leq 2006/07\\ ak,\;\text{for}\;y>2006/07 \end{array}\right.$$ (7)

where *k* and *a* are constant parameters. Alternatively, if there was a growing trend of *R*_y_ in addition to the time-dependent introduction of RT-PCR, the estimated *R*_y_ should better be described by

$$R_{y}=\left\{ \begin{array}{l} k+bt,\;\text{for}\;y\leq 2006/07\\ a(k+bt),\;\text{for}\;y>2006/07 \end{array}\right.$$ (8)

where *b* measures the liner time-dependent trend. Parameterizing *R*_y_ in these simple ways, we compared the AIC between models (7) and (8), identifying better fitted model to the empirically observed data.

### Sensitivity analysis

When the reported outbreak events were classified as foodborne or human-to-human transmissions, a substantial number of outbreak events remained cause “unknown”. Such events accounted for 17.1% to 47.5% of all the reported outbreak events from 2000 to 2016. We conducted the following two types of analysis: (i) we discarded those cause “unknown” events by assuming that the missing data occurred completely at random, and (ii) we added a certain fraction of the cause “unknown” data to the foodborne transmissions and the remaining fraction to the human-to-human transmission events. When we allocated the unknown causes to the two known groups, we varied the proportion to be added to the human-to-human transmission events from 0%–100%.

Another sensitivity analysis was carried out to examine the impact of ascertainment bias on our estimates of *R*_y_, because outbreaks with human-to-human transmissions may have been more likely detected and reported, and it is valuable to quantify the impact of such bias. To do so, we varied the count data of outbreaks, *m*_y_ human-to-human and *n*_y_ foodborne outbreaks, by introducing a factor of ascertainment, *c*. That is, without ascertainment, *c* is assumed to be equal to 1. Nevertheless, if human-to-human outbreaks are *c* times more likely reported, the observed proportion of human-to-human outbreaks should change to *cm*_y_/{(*n*_y_+*m*_y_)(1- *m*_y_/(*n*_y_+*m*_y_)+*cm*_y_/(*n*_y_+*m*_y_)} where *m*_y_ and *n*_y_ are unbiased (actual) numbers of human-to-human and foodborne outbreaks in that adjusting formula. *R*_y_ was then estimated using the biased counts of human-to-human and foodborne outbreaks by varying *c* from 0.5 to 5.0.

### Ethical considerations

The present study reanalyzed data that is publicly available in Japan. As such, the datasets used in our study were de-identified and fully anonymized in advance, and the analysis of publicly available data without identity information does not require ethical approval.

### Supporting data availability

The present study used publicly available data, and the essential components of the epidemiological data were extractable from a website [24] and also S1 Table and S2 Table.

## Results

**Fig 2:**
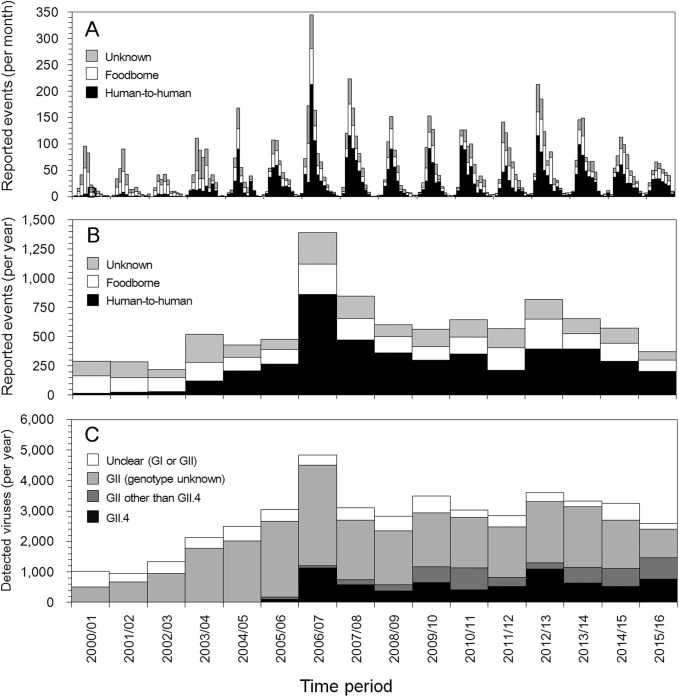
Outbreak events for noroviruses from 2000/01 to 2015/16 in Japan. (A) The reported number of outbreak events from 2000/01 to 2015/16 as monthly data. Every tick mark represents the beginning of September. (B) Outbreak event counts by year. Both (A) and (B) classify the transmission routes as outbreaks caused by human-to-human transmission (black), foodborne infection (white) and unknown causes (gray). (C) The reported number of each detected norovirus genogroup or genotype (detected viruses). White, light gray, dark gray and black colors correspond to unclear (GI or GII), GII (genotype unknown), and GII (except for GII.4 and GII.4), respectively.

Fig 2 shows the time-dependent dynamics of the reported norovirus outbreaks from 2000 to 2016 in Japan. A total of 9,264 outbreaks were reported from 2000/01 to 2015/16 seasons, among which 4,516 were human-to-human, 2,465 were foodborne and the remaining 2,283 were of unknown etiology. Mean and standard deviation (SD) of the reported outbreaks per year were 579 and 280 outbreaks, respectively, and the maximum was 1,388 in 2006/07 and minimum was 220 outbreaks in 2002/03. The minimum after 2006/07 was 373 outbreaks in 2015/16. Every year the peak month for infections was December (Fig 2A). An abrupt increase in the proportion of human-to-human transmission outbreaks is apparent after the 2003/2004 season, the proportion of which peaked in 2006/2007 (Fig 2B). The timing of the increase in human-to-human transmission outbreaks coincided with the time at which GII, and especially GII.4, increased, and this is especially notable in 2006/07 (Fig 2C).

**Fig 3:**
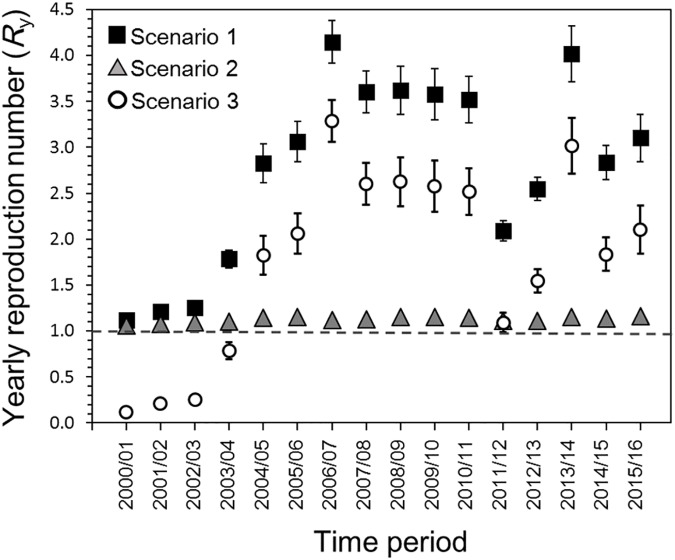
Reproduction number (*R*_*y*_) from 2000/01 to 2015/16 in Japan. The *R*_*y*_s of three different interpretation scenarios are presented. The whiskers for each *R*_*y*_ extend to the upper and lower 95% confidence intervals. The horizontal dotted line corresponds to the *R*_*y*_ at unity (i.e., the value of 1), below which a major epidemic event cannot occur.

Fig 3 shows the *R*_*y*_ estimates based on each interpretation scenario. Under scenario 1, the *R*_*y*_ ranged from 1.11 (95% CI; 1.09–1.14) to 4.15 (95% CI; 3.91–4.38) and peaked in 2006/07. Adopting scenario 2, *R*_y_ was estimated to range from 1.06 (95% CI; 1.06–1.07) to 1.16 (95% CI; 1.16–1.17). Small increases in the estimate occurred in 2005/06, 2013/14 and 2015/16. Under scenario 3, *R*_y_ ranged from 0.11 (95% CI; 0.09–0.14) to 3.29 (95% CI; 3.05–3.52). The estimate exceeded the value of 1 in 2004/05. Employing the trend test, time-dependent increases in estimates of *R*_*y*_ were identified in all three scenarios (p<0.05). Using AIC, scenarios 1 and 3 both appeared to yield the best fit (AIC = 100.3), followed by scenario 2 (AIC = 310.1).

**Fig 4:**
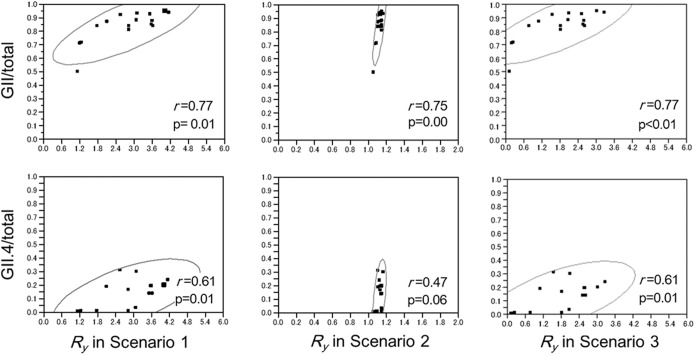
Correlation between the reproduction number (*R*_*y*_) and the proportion of GII or GII.4 among all the detected noroviruses. The top three panels show the correlations between *R*_*y*_ and the proportion of GII (GII.4) among all the detected noroviruses, as based on scenarios 1, 2 and 3, respectively. Similarly, the bottom three panels examine the correlation between *R*_*y*_ and GII.4.

Possible correlations were explored between the estimated *R*_*y*_ value and the GII genogroup and the GII.4 genotype proportions among the total (Fig 4). As we can identify the time-dependent increasing trend in the proportions of the GII genogroup and the GII.4 genotype among the total (p<0.05), a positive correlation was observed between *R*_*y*_ in each scenario and the proportion of GII. A positive correlation was also the case between *R*_*y*_ and the proportion of GII.4, although the correlation in scenario 2 was only marginally significant.

**Fig 5:**
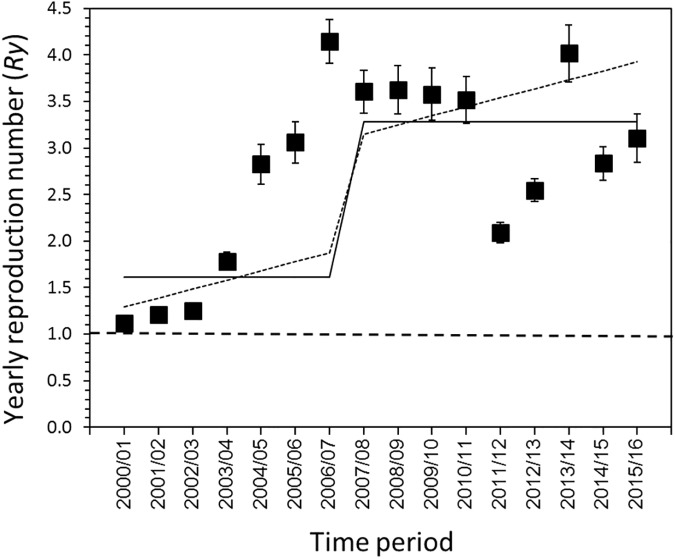
Parametric trend of the yearly reproduction number of noroviruses. Black squares represent the yearly reproduction number from 2000/01 to 2015/16 under scenario 1. The best fit models for the estimated yearly reproduction number with step function alone (solid line) and with step function plus a linear trend (dotted line) are shown. Akaike Information Criterion (AIC) values for solid and dotted lines were 542.5 and 480.7, indicating that the model with linear trend fitted to the data better.

Using parametric models of *R*_y_ as described by Eqs (7) and (8), we examined if observed proportions were biased by time-dependent artefact (e.g. caused by the time-dependent change in the laboratory diagnostic method). Considering that *R*_y_ could be described only by the step function dividing the overall time-dependent pattern into those before and during 2006/07 season and afterwards, AIC values of scenario 1, 2 and 3 were 542.5, 3774.4 and 8793.5, respectively (Fig 5). If we additionally account for the linearly increasing time trend in *R*_y_, the AIC was calculated at 480.7, 3728.9 and 8782.8, respectively, for scenarios 1, 2 and 3. Namely, the model fit was always better for *R*_y_ that combines step function (separating before and after 2007) with linear trend than *R*_y_ with step function only.

**Fig 6:**
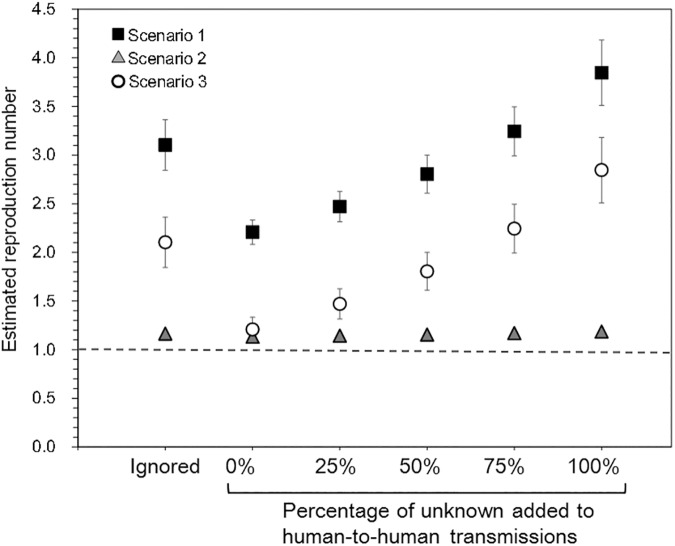
Estimates of the reproduction number of noroviruses in relation to missing data. Black squares, gray triangles, and white circles correspond to the estimated reproduction number in 2015/2016 as based on interpretation scenarios 1, 2 and 3, respectively. “Ignored” results represent the data obtained after discarding the “unknown” cause category datasets. By allocating a certain fraction of “unknown” causes to the human-to-human transmission outbreaks, the relative changes in the estimated reproduction number could be explored. Whiskers extend to the upper and lower 95% confidence intervals.

As part of the sensitivity analysis, Fig 6 explores the impact of the cause “unknown” category data on our estimate of *R*_y_. As one can imagine, the estimate of *R*_y_ would be greater than *R*_y_ when we discarded unknown data. The scenario-related estimates order (Fig 6) and the time-dependent patterns of *R*_*y*_ remained unchanged, even when we assigned a certain fraction of the “unknown” causes to the human-to-human transmission events.

**Fig 7:**
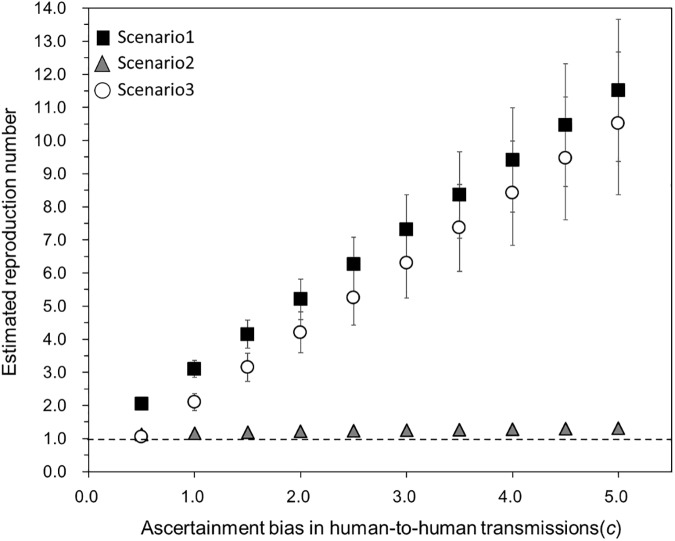
Estimates of the yearly reproduction number of noroviruses in relation to the ascertainment of human-to-human transmission outbreaks. Black squares, gray triangles, and white circles represent the yearly reproduction numbers in 2015/16 season in our interpretation scenarios 1, 2, and 3, respectively. Whiskers extend to the upper and lower 95% confidence intervals. We set the factor of ascertainment *c*, i.e. the relative frequency of human-to-human outbreaks to be reported compared with foodborne outbreaks, as a coefficient that represents the ascertainment bias. Observed frequency data in 2015/16 season were assumed as unbiased and *c* = 1.

Using the empirical data in 2015/16 season, Fig 7 explores the impact of ascertainment bias of human-to-human outbreaks on the estimates of *R*_y_. Overall, *R*_y_ appears to be overestimated if the ascertainment bias is large. Among three scenarios, scenarios 1 and 3 yielded *R*_y_ that are more sensitive to the increase in ascertainment factor *c* than scenario 2. In scenarios 1 and 3, increase of *c* by 1.0 led to an increase in *R*_y_ by 1.05.

## Discussion

The present study estimated the *R*_*y*_ for NVIs from 2000/01 to 2015/16 by assessing their time-dependent patterns and exploring the possible association between *R*_y_ and the norovirus genotype. Since the data generating process cannot fully ascertain the mode of transmission, the corresponding uncertainties were addressed by employing three possible scenarios to interpret the data. An apparent increase in *R*_*y*_ (with small fluctuations) was observed under all the scenarios, along with positive correlations between *R*_*y*_ and the proportions of GII and GII.4 among the total number of viruses identified. The increasing pattern of *R*_y_ was better captured by a simple model with a linear trend plus the step function effect of introducing RT-PCR to laboratory diagnosis than a model with step function alone, implying that the increase in *R*_y_ was not only caused by the widespread introduction of RT-PCR at one time. To the best of our knowledge, the present study is the first to successfully describe the time-dependent change in NVI transmissibility using empirically observed epidemiological data.

Growth in norovirus transmissibility over time implies that the infection has become a more human-to-human transmissible disease. This finding was achieved by exploring the long-time series data from 2000 to 2016, and the time-dependent change indicates that NVI in the early period may differ somewhat from the time-dependent changes seen in recent infections. For instance, foodborne cases of NVI arising from bivalve molluscan shellfish (raw oysters) have decreased relatively as a fraction among the total number of infected individuals in Japan [27], while noroviruses better fitted to humans have selectively caused the human-to-human transmissions, thereby changing the epidemiology of this pathogen. Our order of 3 to 4 estimates for *R*_*y*_ in recent years is consistent with the assumed values of the basic reproduction number *R*_0_ in a previously published study [20]. The comparability of *R*_y_ to *R*_0_ and its time dependency endorse its utility to monitor strain specific transmissibility over time in the future.

Moreover, the increased time-dependent pattern of the *R*_*y*_ appears to have coincided with an increase in the proportions of GII and GII.4 genotypes (especially in 2006 when the GII.4 2006b strain was identified [28–30]), implying that the viral genogroup or genotype is responsible for characterizing the increased transmissibility. The positive association between transmissibility and GII is consistent with the findings of a published systematic review [31]. While GII.4 was not linked to transmissibility in the same report [31], another study showed that GII.4 is likely to be involved in human-to-human transmissions [8, 32, 33]. GII.4 is known to bind to histo-blood group antigens more than other genotypes, and the diversity of its P2 domain implies that this genotype may experience more rapid molecular evolution than other genotypes [6,7,34]. Two studies have indicated that while transmission of GII.4 2006b has decreased [35, 36], it has been replaced by later genotypes such as GII.4 New Orleans 2009 and Sydney 2012, and new GII.4 variants may likely replace the earlier GII.4 genotype because substantial herd immunity to earlier genotypes probably exists [37–39]. The present study has contributed to improved understanding of the evolutionary dynamics of NVI by demonstrating that the time-dependent infection dynamics can be partly explored by estimating the *R*_y_ and examining the epidemiological and virological data concurrently. Continued monitoring of the major routes of transmission for NVIs is of the utmost importance, while better understanding of the mechanisms underlying norovirus evolution is eagerly awaited.

Scenarios 1 and 3, which rely on the random sampling of outbreaks or the random distinction of sporadic cases without any offspring from other series of cases, appeared to fit to observed data equally well in statistical sense. While our estimates of *R*_y_ in recent years were consistent with those published in the USA [20], it must be noted that the use of proportional data for human-to-human transmission among the total number of reported cases would inherently face ascertainment and reporting biases. That is, compared with sporadic cases, cases that are attributable to human-to-human transmission are more likely to be diagnosed and are also more likely to be notified, as has been noted in modeling studies that have focused on the human-animal interface [40,41]. In fact, one prefecture arbitrarily but descriptively defined a human-to-human transmission outbreak as an outbreak in various facilities where more than 10 cases a day or more than half of all residents developed severe symptoms [37]. We have thus conducted a sensitivity analysis, quantifying the impact of ascertainment bias on *R*_y_ estimates (Fig 7). Our *R*_y_ may have been potentially overestimated, influencing any policy relevant decisions derived from the magnitude of *R*_y_ (i.e. absolute value of *R*_y_), but the relative change in *R*_y_ over time is likely to be unaffected by ascertainment and reporting biases.

As mentioned in Methods, foodborne outbreaks have been frequently differentiated from human-to-human outbreaks due to the need to link the outbreak to a source of infection (e.g. a shared food item) among cases. Cases in the foodborne outbreaks are likely tested, because public health needs to verify that a particular foodborne outbreak was caused by novoviruses, and in that sense, the environmental exposure event can be regarded as well identified in the empirical observation. Rather than the validity of environmental exposures, we have not been sure as to the data generating process of “unknown” category data. We have thus conducted a sensitivity analysis in Fig 6 and the time-dependent change in *R*_y_ remained similar to Fig 3.

It should be noted that we employed three possible scenarios to address the uncertainty surrounding the interpretation of the mode of transmission. Scenario 1 assumed random sampling of all outbreaks, while it is likely that large epidemics may be easily reported compared with small outbreaks. Scenario 2 assumed random sampling of cases, while in reality more severe cases are likely reported than mild ones. Scenario 3 assumed that the presence of one secondary transmission as human-to-human outbreak, but it does not capture the tendency that the presence of two or more secondary transmissions lead to greater ascertainment than only one secondary transmission. While it is evident that each did not fully capture the observed patterns of the data, we were not able to exclude any scenario as long as each could theoretically lead to the observation of empirical data in Japan, and thus, we believe that employing three models and comparing estimates were the best practice to address the uncertainty surrounding the mode of transmission.

Four other limitations should be noted. First, technical changes in laboratory methods, reporting rules for NVIs and any other time events were not explicitly taken into account for the 16 year study period. As mentioned in Methods, the widespread use of RT-PCR in and after 2007 was one important factor that could have contributed to increased proportion of reported GII.4 cases from around 2006. We have thus modeled *R*_y_ using a step function with and without linear trend, and it was shown that the step function with linear trend was favored. Nevertheless, it should be remembered that the step function qualitatively captured the increase in *R*_y_ in any case, and we cannot exclude the possibility that RT-PCR had an impact on *R*_y_. At least, we have shown that there was a linearly increasing trend that has not been explained by the introduction of single laboratory technique. Other reasons, e.g. disproportionally improved surveillance of human-to-human transmission especially at healthcare facility, cannot be excluded, and indeed, we have shown that an improved ascertainment would lead to an increase in *R*_y_. Nevertheless, we do not have any explicit indication of such disproportionate improvement as compared to published studies on the relationship between the transmissibility and GII or GII.4 [8,31–33], and clarification on such alternative explanations would be the subject for future studies.

As the second limitation, by grouping all noroviruses in a single epidemic season into one category our analysis rests on an estimation of the *R*_y_. While the present study explored possible correlations between *R*_y_ and the proportion of certain genogroups and genotypes, an aggregated analysis had to be adopted because of the unlinked nature of the empirical data between each single outbreak event and the specific genogrouping and genotyping. Third, our estimation did not explicitly account for herd immunity. Only by assuming that *R*_y_ reflects both the transmissibility and susceptible fraction of the population could a homogeneously defined estimate be derived from the data. The ignorance did not allow us to decompose *R*_y_ into the actual transmissibility of a particular year *y* and a possible partially immune fraction in the same year. Only by collecting more structured data (e.g. an age-structured long-time series combining the transmission route and virus genogrouping), would we be able to explicitly address the issue of herd immunity, and *R*_y_ estimates presented here might have been potentially overestimated. Fourth, we did not account for any index cases arising from (other) unrecognized chains of transmission. Given that such index cases exist, the estimate of *R*_y_ could be greater than what have been estimated in this article.

In summary, a time-dependent increase in NVI transmissibility was identified in Japan, indicating that noroviruses better fitted to humans have selectively caused the human-to-human transmissions. This increase in norovirus transmissibility has coincided with an increase in the proportions of GII and GII.4 among all the viruses detected, implying a possible association between transmissibility and this genotype and genogroup. Evolutionary dynamics are perhaps responsible for the changing epidemiology of noroviruses, thereby elevating the relative importance of human-to-human transmission as compared with classical foodborne infections. We believe that our findings will greatly enhance current understanding of the ecoepidemiology of noroviruses. However, we cannot help but stress the importance of continued monitoring of the fraction of human-to-human transmission outbreaks among the total outbreak events to better capture the evolutionary nature of norovirus and investigate effective control strategies against it.

## Supporting information

S1 Methods (DOCX)

S1 Table (XLSX)

S2 Table (XLSX)
