# Supplementary material for: The transmissibility of noroviruses: Statistical modeling of outbreak events with known route of transmission in Japan
Source: PLoS One. 2017 Mar 15;12(3):e0173996. doi: 10.1371/journal.pone.0173996 (PMC5352013; doi:10.1371/journal.pone.0173996)
Supplement: S1 Methods — (DOCX) [file pone.0173996.s001.docx]

**S1 Methods**

The transmissibility of noroviruses: Statistical modeling of outbreak events with known route of transmission in Japan

Ryota Matsuyama, Fuminari Miura, Hiroshi Nishiura

**1. Equation 2 for scenario 2:**

Equation 2 is composed of two parts: One is the expected number of infected cases in a branching process (i.e., *R_y_* /*R_y_*-1), and the other is related to normalization by population size (1/*N*). The former is derived from the probability generating function of the final size *G*_z_(*s*) which satisfies

$G_{Z}(s)=\frac{s}{1+R_{y}(1-G_{Z}(s))}$, (A1)

the derivative of the solution of (A1) and yields the final size distribution. The expected outbreak size for *R*_y_>1 requires the calculation of Pr(*Z*=*z*)/*π* where *π* is the probability of extinction (*π*=1/*R*_y_) [25]. Hence, we have

$z_{minor}=\frac{R_{y}}{R_{y}-1}$ (A2)

**2. Equation 5 for scenario 2:**

We employed the final size equation of the homogeneous version of the Susceptible-Infectious-Removed (SIR) model. Namely, we adopt

$1-z_{major}=\text{ }\exp(-R_{y}z_{major})$ (A3)

We approximate the right-hand side of (A3) using the Taylor series,

$\begin{matrix} \exp(-R_{y}z_{major})=1+\frac{-R_{y}z_{major}}{1!}+\frac{\left(-R_{y}z_{major}\right)^{2}}{2!}+\frac{\left(-R_{y}z_{major}\right)^{3}}{3!}+\cdots\\ \approx1-R_{y}z_{major}+\frac{1}{2}R_{y}^{2}z_{major}^{2} \end{matrix}$ (A4)

Consequently, the final size satisfies

$z_{major}\left(z_{major}+\frac{2\left(1-R_{y}\right)}{R_{y}^{2}}\right)=0$, (A5)

thereby leading to the solution in the main text.

**3. Probability of extinction within one generation in scenario 3:**

We assume that the offspring distribution follows a geometric distribution that has a probability mass function,

$g(k)=\frac{1}{1+R_{y}}\left(\frac{R_{y}}{1+R_{y}}\right)^{k}$ (A6)

The probability of extinction without any secondary case is equal to *g*(0), leading to the likelihood function in the main text. It should be noted that an advantage of (A6) is that we do not have to assume that *R*_y_>1 or *R*_y_<1.
